# Bending the Arc towards Equitable Partnerships in Global Health and Applied Training

**DOI:** 10.5334/aogh.2564

**Published:** 2019-11-06

**Authors:** Nina A. Martin, Anna Kalbarczyk, Emily Nagourney, Abigail Reich, Bhakti Hansoti, Andrew Kambugu, Thomas C. Quinn, Yukari C. Manabe, Barbara Castelnuovo

**Affiliations:** 1 Johns Hopkins Center for Global Health, Baltimore, MD, US; 2 Department of International Health, Johns Hopkins Bloomberg School of Public Health, Baltimore, MD, US; 3 Department of Emergency Medicine, Johns Hopkins University School of Medicine, Baltimore, MD, US; 4 Infectious Diseases Institute, Makerere University College of Health Sciences, Kampala, UG; 5 National Institute of Allergy and Infectious Diseases, National Institutes of Health (NIH), Baltimore, MD, US; 6 Division of Infectious Diseases, Department of Medicine, Johns Hopkins University School of Medicine, Baltimore, MD, US

## Abstract

**Background:**

Global health education has rapidly expanded in popularity, and many programs require applied practical experiences. Applied experiences are critical for global health training. Often a trainee from a high-income country travels to work with collaborators and partners in a low- or middle-income country. These experiences exist within partnerships between individuals and institutions that have varying objectives, including research, program implementation, or education. Attention is growing to ensure equity in these relationships in ways that are informed by the voices of collaborators and partners.

**Objectives:**

Understanding the experiences of LMIC collaborators in academic global health partnerships is essential. Our research aimed to capture views of our partners about factors impacting equitable global health partnerships.

**Methods:**

We conducted a small survey among global health collaborators and partners who host students on these experiences. Respondents were asked to rank enablers and barriers to equitable partnerships in priority order. Results were stratified by institutional affiliation and role.

**Results:**

Funding, time, engagement, and mutual opportunities for training are common enablers and barriers of global health partnerships. There were slight differences across different professional roles. Other reported factors that impact partnerships included language barriers, visa concerns, and identifying opportunities for collaboration.

**Conclusions:**

Our work highlights several barriers and enablers faced by partners that align with those reported across the global health education community. Equitable partnerships are possible and require substantial input at individual, interpersonal, and institutional levels. We reflect on two strategies to encourage partnership equity employed within our own work and discuss how these strategies can be applied more broadly.

## Introduction

Robust global health training hinges on quality field experiences. Many academic global health programs include practicums in their degree curricula. Field experiences provide an opportunity to strengthen classroom-based learning with experiential, and can take the form of research projects, clinical practice electives, or education-based service delivery experiences. The quality and impact of the experience is highly dependent on the efforts and commitment of both sending institutions and in-country hosts.

The Johns Hopkins Center for Global Health (CGH) funds almost 100 undergraduate, graduate, and medical trainees for global field experiences annually [1]. In the context of our most popular programs, a trainee develops a project based in a low- or middle-income country (LMIC) with a high-income country (HIC)-based faculty mentor and in-country LMIC collaborators. The trainee travels to the LIMC field site to work for a minimum of six weeks. Depending on the site’s maturity and size, on-site mentorship is provided by a spectrum of professionals including coordinators, investigators, department heads, local peers, or even expatriates from the home institution who are based in the LMIC. These experiences are usually organized within existing inter-institutional partnerships. One such example is the 18-year partnership between John Hopkins University and the Infectious Diseases Institute (IDI) at Makerere University in Uganda. Starting as an alliance between Ugandan and North American researchers to transform HIV care, IDI is now recognized as a leading independent research and training center that fosters inter-institution capacity strengthening and mutual advancement [2]. Annually they host trainees globally for training in clinical skills, laboratory science, research, population health, and implementation science.

Recent literature has highlighted the disproportionate burden that LMIC partners face in supporting field experiences, particularly in relation to the perceived benefit, and the human resources needed to support them effectively [3,4,5,6,7]. Additionally, these partnerships can result in disproportionate benefits for the HIC institutions who may gain more opportunities for prominent authorship positions, conference presentations, and funding. Factors driving inequitable partnerships include lack of trust between partners, organizations, or institutions, lack of transparency in communication between partners, or high-level structural elements [8,9]. Hedt-Gauthier et al. highlight several ways in which HIC academic promotion requirements are a major cause of partnership inequities [10]. They note defined academic promotion tracks that Prioritize “publications, grant funding, and reputation, the latter generally assessed by the number of conference presentations”. Of several factors, the group emphasizes that the lack of consideration of first or senior LMIC authorship on key publications, no assessment of the quality of HIC engagement with LMIC partners, and lack of valuation of substantial HIC faculty time spent in an LMIC alongside collaborators during promotional review ultimately disincentivizes HIC faculty from building equitable partnerships.

There is a growing importance on understanding the views of LMIC collaborators in academic global health partnerships [9,11]. In the context of global research, we view equitable engagement with partners as mindful response to relationship dynamics, values, and resources described by Walsh et al. [12] The research reported in this paper seeks to add to the growing body of international collaborators’ views on how to make global health partnerships more equitable and mutually beneficial.

## Methods

### Survey design

The study team developed an online survey to determine international collaborators’ views on barriers and facilitators to equitable partnerships. Informal conversations with mentors and collaborators in both HICs and LMICs informed the first iteration of questions. These were revised to incorporate common barriers and enablers identified in published literature, and responses submitted by trainees to routine CGH program evaluations. The initial survey contained 17 questions; three questions (key screening, demographic, and ranking questions) were required. Survey questions captured demographic information and asked respondents to rank in priority order 12 enablers and barriers to academic global health partnerships. A rank of ‘1’ indicated high importance, increasing rank indicated lesser importance. Space was provided for respondents to indicate additional barriers and enablers. As additional responses were provided, study team members added 3 new barriers and enablers to the list for a new total of 15 items, in order to reflect emerging factors. Questions were finalized through discussion and consensus among study team members.

### Study population

Survey respondents were recruited in-person and via email, sampled from members of the Consortium of Universities for Global Health (CUGH) and attendees of their 9^th^ annual conference “Health Disparities: A Time for Action” in March 2018 [13]. Founded in 2008, CUGH includes over 170 member institutions from around the world engaged in global/public health training and research. The study team attended the 9^th^ annual CUGH conference (New York, NY, USA) and identified potential respondents who were based at LMIC institutions during conference talks and events. Participants were eligible to complete the survey if they answered “yes” to both screening questions: 1) “Do you spend the majority of your time (>7 months) per year in an LMIC setting?” and 2) “Does your institution or organization host students from HICs?” Ineligible participants were thanked for their time and directed away from the survey. Eligible participants were routed to the remaining survey questions.

### Data collection and analysis

Potential respondents were approached by the study team and given a card which briefly described the study’s purpose and a link to the survey. These cards were left in conference meeting rooms and exhibition booths for further distribution. The survey was built in Qualtrics^©^ (Qualtrics, Provo, UT) and distributed after the conference via the CUGH Education listserv, which reaches approximately 1,547 CUGH members involved in trainee education or support.

All responses were exported, corrected for spelling and formatting, cleaned, and analyzed in Microsoft Excel^©^ (Microsoft Corporation, Redmond, WA). Role was self-reported from four choices: Student/Researcher/Laboratory science support, Program Coordinator/Administrator, Faculty/Assistant Professor/Associate Professor, and Dean/Professor. Institutional affiliation was also self-reported by respondents in free response and later categorized by study staff as non-governmental organization, university, hospital/health center, research organization, public/private agency, government, or not disclose. Incomplete responses were removed from the analysis. The mean rank was calculated across responses and stratified by institution and role. Open-ended responses were cleaned and condensed by study staff.

### Ethical review

The study protocol and survey design were reviewed and approved by the Johns Hopkins Medicine Institutional Review Board (IRB #164921). At the start of the survey, participants were provided information about the survey, including potential benefits and risks. Participants were required to agree to participate to continue with the survey. Those who did not agree automatically exited the survey.

## Results

One hundred and sixty-six people opened the survey, of which 156 (94%) agreed to participate. Of those, 99 of 156 (63%) met the first screening criterion (spent more than 7 months per year in an LMIC) and of those, 65 (67%) met the second screening criterion (reported hosting students from high-income countries). Nineteen provided no responses after eligibility was assessed. A total of 46 respondents met all inclusion criteria and were included in the analysis. Respondents spent time in 26 different LMIC countries, with the majority of respondents in sub Saharan Africa (31, 67%). Eleven (24%) were female and 34 (74%) were male; 1 (2%) declined to share their gender. Ten (22%) reported they were a “Dean/Professor”, 17 (37%) were Faculty/Assistant or Associate Professors, 11 (24%) were Program Coordinators or Administrators, and 8 (17%) were students, researchers, or laboratory support. Respondents’ institutional affiliations included universities (22, 48%), hospitals/health centers (10, 22%), non-governmental organizations (7, 15%), research organizations (3, 7%), public/private agencies (1, 2%), government (1, 2%), or did not disclose (2, 4%). Forty-five respondents (98%) provided complete ranking information in addition to demographic information; analysis of their responses is detailed below.

Tables 1, 2, 3 show heat maps of enablers and barriers identified by participants overall and stratified by institutional affiliation and role. Across all responses, the top ranked enablers to equitable partnerships in student training (in order of most important to least) were 1) having a US partner actively involved in education/research in LMIC setting, 2) having funding from a US institution, and 3) opportunities for LMIC health professionals to come to the US for training and exchange programs. Top ranked barriers were 1) lack of funding, 2) the short length of student elective/experience, and 3) lack of engagement by US partner. Across all institutional affiliations, funding and partner engagement, or lack thereof, were ranked most important. “Earlier professionals” (those identifying as students, researchers, or coordinators) prioritized funding as a key enabler to effective partnerships as compared to “senior personnel” (those identifying as faculty members or Deans), who viewed partner engagement as more important (Table 3). All groups except students, researchers, and laboratory scientists reported training opportunities between the US and LMICs as an important enabler to successful partnerships.

**Table 1:**
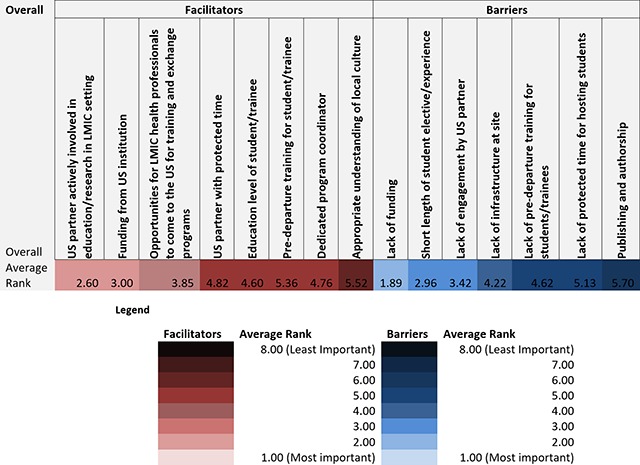
Average ranking of facilitators and barriers overall.

**Table 2:**
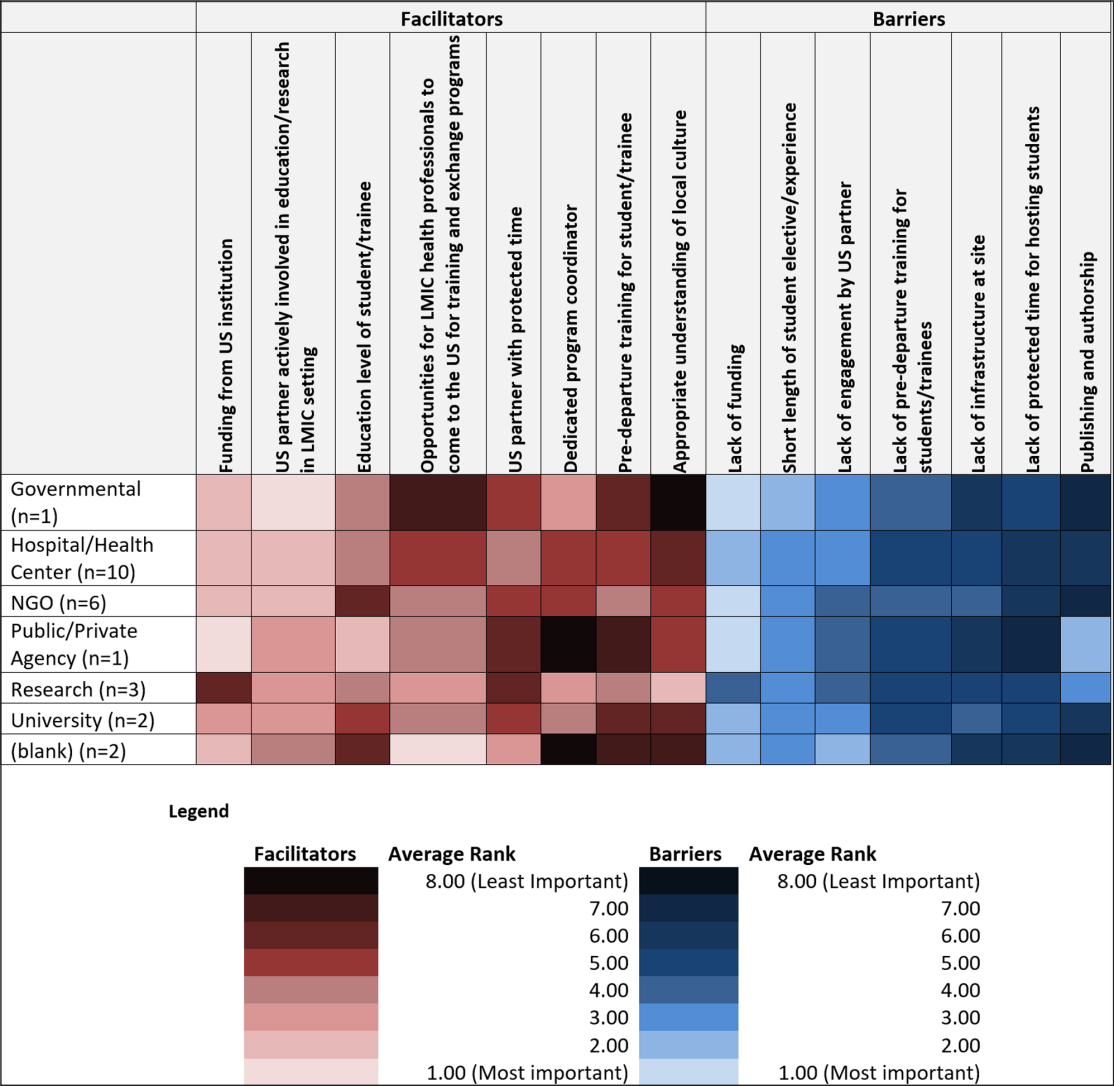
Average ranking of facilitators and barriers by Institutional Afiliation.

**Table 3:**
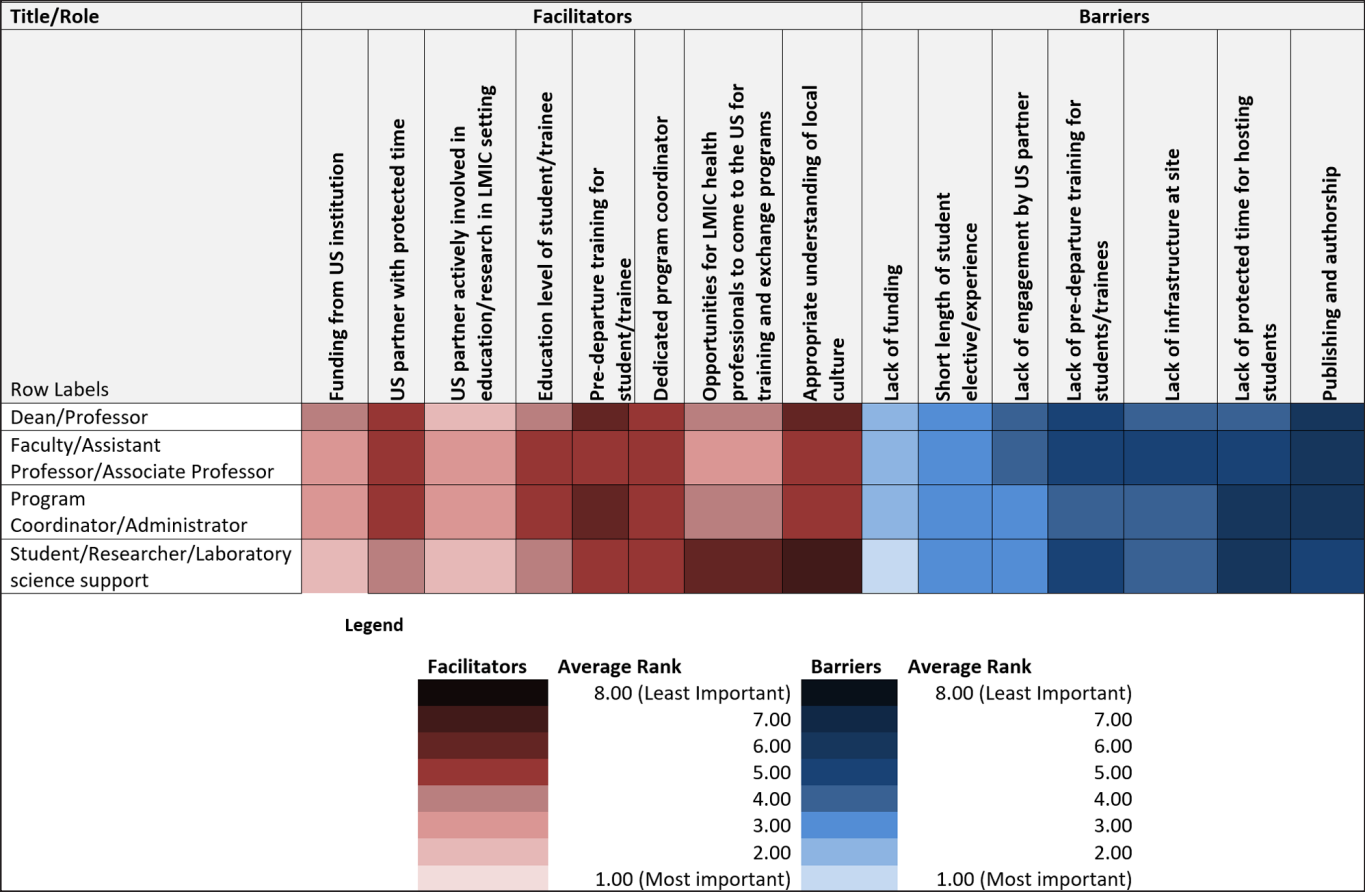
Average ranking of facilitators and barriers by title/role.

Across all groups and strata, lack of funding and lack of engagement by US partners were ranked as important barriers to partnerships. Other barriers listed in open-ended response section of the survey included language barriers, immigration and visa concerns, lack of “dissemination of opportunities for institutional partnership”, burdensome students, and “power relationship[s] between institutions”.

## Discussion

Equity in partnerships is not a new concept. Many investigators in organizational development, education, and other fields strive to understand how to promote mutual success in a collaborative environment. Many of the barriers reported here continue to be critical obstacles that, to our knowledge, no existing model has comprehensively addressed. The responses we received resonate with our authors, who collectively have over six decades of global health experience as mentors, leaders, and trainees themselves at overseas institutions.

From our respondents, funding, time, engagement, and opportunities rose to the top as critical enablers and barriers of academic global health partnerships. As barriers, lack of funding, insufficient time, and improper investment in training and long-term planning can cripple the most well-intentioned projects. While we do not have the depth in this survey to delve further, the slight divide between senior and more junior personnel aligns with our collective experiences. Funding is a critical component of a young person’s career, particularly in academic settings. Establishing funding relationships and work portfolios that are appealing to funders is a concern for more junior investigators. Senior personnel may already possess a history of funding, professional networks, and mentorship relationships which facilitate the process of seeking and applying for funding to manage their institutes, programs, and research portfolios. As enablers, steady funding streams provide stability to allow continued progression of work overtime. Strong engagement between HIC and LMIC partners allow for the development of trust, which is essential for any productive interpersonal interaction. Commitment to supporting opportunities for local research capacity building can shift a partnership’s focus towards mutually beneficial engagement and commitment to the partnership’s outcomes. The introductory example of IDI is one example of how these enablers can come together to affect future success. A generous contribution from Pfizer coupled with forward-thinking leadership focused on capacity building and investment in training has led to the expansion of the IDI partnership into a sustainable, trusted institution.

One limitation of our survey is that questions originated largely from barriers observed in the literature as well as informal reflections from key informants. Future iterations should build on the results of additional interviews to explore and adjust item phrasing and connotation. Future surveys should randomize the order of ranking items to minimize the risk of selection bias. We added three emergent factors to the list of potential barriers partway through survey administration, limiting the number of potential respondents to all response options. We recruited a limited sample from a largely academic global health-oriented pool whose participants likely represent those with the resources to travel, restricting generalizability. Additionally, the questions focus on global health education training, and so cannot be extrapolated to other fields that may face similar or distinct barriers. Widening the sample base and allowing for supplemental focused qualitative assessments could lend insight on additional important barriers and enablers.

Equity carries heightened importance when considering the historical evolution of global health. We must acknowledge that many countries considered low- or middle-income were former colonies, subjected to disastrous economic and social policies, with whose long-term effects many nations are still grappling. Global health has been criticized for replicating a neocolonial architecture and reinforcing power systems that favor HICs under the guise of social good [14,15,16]. How do we change the narrative of global health training partnerships towards equity? First, there should be a wider exploration of the key barriers and enablers of equitable partnership that includes ongoing dialogue between HICs and LMICs. Our report provides one snapshot of challenges and enablers faced by a subset of global health practitioners. Student elective length was reported as an important barrier to effective training partnerships. While not always possible, longer training experiences should be encouraged. At CGH, we enforce a minimum time requirement of six weeks for almost all student electives because we believe anything short of that does not allow for true relationship building and contribution. Training and capacity strengthening are critical building blocks of systems working towards equity [14,17]. One avenue to support equitable training is to increase the number of opportunities available for LMIC partners to come to HICs for mentorship, such as the Fogarty Global Health Fellows Program [18]. The resources to support quality electives should not be overlooked. CGH’s Global Established Multidisciplinary Sites (GEMS) program provides financial support for in-country partners to fund the facilitation, management, and administration of student experiences [19]. The AMPATH consortium is another model to address the underlying issues of time and funding [20]. Founded in 1990 between Moi University and Indiana University, the consortium has expanded to include several university members and requires a longer term (usually 1.5–2 years) in-country residency, co-training with local practitioners, and a substantial financial contribution from each member institution.

Second, best practices to foster equity can be made the standard of practice. Many conversations on how to do this in a way that engages HIC and LMIC institutions have already started. Several tools such as the Partnership Assessment Toolkit or the Partnership Analysis tool exist as practical checklists to articulate expectations and evaluate partner engagement [21,22]. Academic journals can be encouraged to address the need for diversity in editorial boards, author gender, and author geography [23,24,25,26]. At CGH, we can better foster a social justice-oriented dialogue about partnership inequity with our trainees [27]. The next phase of our research is to explore additional barriers and enablers of Johns Hopkins’s partnerships with academic training institutions. We plan to engage different consortia of Universities and research institutions, such as AFREhealth, to determine broader best practices for building equitable partnerships in this new age of global health. We hope that if our trainees learn within a system that prioritizes partnership equity, those lessons will permeate future global health work.

The current growing student enrollment trends tell us that global health education in HICs will continue to rise in popularity and scope. Advances in science and technology will continue to further what is possible to achieve good health. Our global health community has a responsibility to ensure responsible, bi-directional engagement regardless of national or institutional affiliation. Such action is in line with the missions of the authors’ respective centers and institutes, as well as the concept that equity, like health, is a right for all.
